# Cultural Differences in Inhibitory Control: An ALE Meta-Analysis

**DOI:** 10.3390/brainsci13060907

**Published:** 2023-06-03

**Authors:** Gioele Gavazzi, Chiara Noferini, Viola Benedetti, Maria Cotugno, Fabio Giovannelli, Roberto Caldara, Mario Mascalchi, Maria Pia Viggiano

**Affiliations:** 1 Department of Neuroscience, Psychology, Drug Research and Child’s Health (NEUROFARBA), University of Florence, 50135 Florence, Italy; gioele.gavazzi@unifi.it (G.G.); noferini@lens.unifi.it (C.N.); viola.benedetti@unifi.it (V.B.); maria.cotugno1@stud.unifi.it (M.C.); fabio.giovannelli@unifi.it (F.G.); 2 European Laboratory for Non-Linear Spectroscopy, University of Florence, Sesto Fiorentino, 50019 Florence, Italy; 3 Eye and Brain Mapping Laboratory (iBMLab), Department of Psychology, University of Fribourg, 1700 Fribourg, Switzerland; roberto.caldara@unifr.ch; 4 “Mario Serio” Department of Experimental and Clinical Biomedical Sciences, University of Florence, 50135 Florence, Italy; mario.mascalchi@unifi.it

**Keywords:** culture, cultural differences, Western culture, Eastern culture, inhibitory control, right prefrontal cortex, meta-analysis

## Abstract

Culture greatly influences our attitudes, beliefs, and behaviors, affecting how we communicate and make decisions. There is an ongoing debate regarding the belief that people from Eastern cultures possess greater self-control abilities when compared to people from Western cultures. In this study, we conducted a meta-analysis using the Activation Likelihood Estimation (ALE) algorithm to compare 30 studies (719 subjects, 373 foci) that used fMRI to investigate the performance in Go–Nogo and Stop Signal Tasks of participants from Western and/or Eastern countries. Our meta-analysis found differences between the networks activated in Eastern and Western culture participants. The right prefrontal cortex showed distinct patterns, with the Inferior Frontal gyrus more active in the Eastern group and the middle and superior frontal gyri more active in the Western group. Our findings suggest that Eastern culture subjects have a higher tendency to activate brain regions involved in proactive inhibitory control, while Western culture subjects rely more on reactive inhibitory brain regions during cognitive control tasks. This implies that proactive inhibition may play a crucial role in promoting the collective and interdependent behavior typical of Eastern cultures, while reactive inhibition may be more important for efficient cognitive control in subjects of Western cultures that prioritize individualism and independence.

## 1. Introduction

Culture is a significant factor in shaping our attitudes, beliefs, and behaviors, impacting everything from our communication styles to our decision-making processes and perceptions of the world. While it is evident that individuals from different cultures exhibit inhomogeneous and sometimes peculiar behaviors and cognitive processes, the underlying neural mechanisms behind these differences remain largely unknown. Although studies have explored cultural differences in various psychological processes, it is still unclear whether these differences are governed by the same or different neural networks. However, research using functional magnetic resonance imaging (fMRI) has identified multiple neural circuits that are involved in different psychological processes [1,2].

It is commonly believed that individuals from Eastern cultures have a greater capacity for inhibitory control than those from Western cultures, and this idea is a subject of ongoing debate. Research in cultural psychology indicates that there are differences in inhibitory control between individualistic, as are typically considered Western cultures, and collectivistic, as are typically considered Eastern cultures [3,4,5,6,7]. This is likely to be associated with the Eastern philosophy, which conveys the message of the importance of self-control and inner discipline that underlies a collectivist type of culture, where the group and society are very important.

The basic idea is that what is good for society is also good for the individual, and a balance between individual and society is achieved by modulating self-control through inhibitory abilities [8,9,10]. From an early age, Eastern education systems adopt strict parenting and parenting practices that are believed to stimulate and strengthen inhibitory control [11,12,13]. Indeed, in studies conducted during early childhood, children aged three to four years old from East Asian nations often demonstrate greater proficiency in measures of inhibitory control, including the Stroop task, when compared to their American counterparts [6,14]. Although it is likely that the difference between people of Western and Eastern cultures may be related to the extent and type of brain regions activated for self-control, when one compares fMRI studies in subjects belonging to the two types of culture, there is a lack of consistency in the neural areas responsible for the respective inhibitory control [15,16,17,18,19].

This inconsistency can be attributed to various factors and differences, including the chosen paradigms. In fact, while inhibitory control has been mainly assessed using the Go–Nogo and Stop Signal Tasks interchangeably, these two tasks seem to tackle differently inhibitory components [20,21,22,23,24,25,26,27,28,29,30].

There are other relevant factors worth taking into consideration, such as the participants’ ages [31] or education levels [32]. Notably, to control for education, it may be useful to also control for cultural proficiency. Inadequate cultural knowledge and/or adaptation have been linked to decreased performance in tasks that require inhibitory control, such as the Wisconsin Card Sorting Test [33] and the Stroop test [34,35]. Conducting a meta-analysis on the data obtained from previous studies could better clarify the implications of the various circuits in the modulation of cognitive control as a function of the two cultures. As far as we know, no meta-analysis has been carried out to investigate the potential variations in neural correlates associated with inhibitory control in Western and Eastern cultures. Thus, the aim of this study is to investigate the influence of Western and Eastern cultures on the neurobiological underpinnings of inhibitory control while controlling for variables that may affect inhibition, such as task design [20], aging [31], and education [32].

To this end, we conducted a meta-analysis and compared 30 studies that used fMRI to investigate the Go–Nogo and Stop Signal Tasks in Western and/or Eastern participants using the Activation Likelihood Estimation (ALE) algorithm. Since our primary goal was to explore the brain mechanisms underlying inhibitory control in these cultural contexts, rather than focusing on the differences in behavioral outcomes, we took steps to minimize confounding effects by exclusively analyzing volumes derived from trials where inhibition was successfully achieved and by carefully matching the two groups based on the task design.

## 2. Materials and Methods

### 2.1. Search Strategy and Selection Criteria

We conducted a systematic and comprehensive literature search to select relevant fMRI studies published up to April 2022 using the databases *PubMed* (https://pubmed.ncbi.nlm.nih.gov/—accessed on 8 April 2022) and *Web of Science* (https://webofknowledge.com—accessed on 8 April 2022). The selected keywords were combined using the Boolean operators AND and OR. The *PubMed* search input was: (“Stop-Signal Task” OR “Stop Signal Task” OR “Go-NoGo” OR “Go NoGo” OR “GoNoGo” OR “Go/NoGo” OR “Go No-Go”) AND fMRI. The *Web of Science* search input was: TS = ((“Stop-Signal Task” OR “Stop Signal Task” OR “Go-NoGo” OR “Go NoGo” OR “GoNoGo” OR “Go/NoGo” OR “Go No-Go”) AND fMRI). Additional studies were searched from the references of all identified publications. Eligibility was determined via a two-step procedure performed by four of the authors (GG, CN, VB, and MC). First, the titles and abstracts of all identified articles were screened. In the second step, the full texts of studies, according to predefined eligibility criteria, were independently examined, and agreement was reached after discussion. Our study was conducted following the preferred reporting items for systematic reviews and meta-analyses (PRISMA) guidelines [36].

The studies were considered eligible if they met the following inclusion criteria: (1) whole-brain analysis performed on fMRI data (we excluded studies conducted using positron emission tomography and fMRI in which only results from ROI analysis were reported); (2) availability of coordinates of activation foci clearly provided either in Montreal Neurological Institute (MNI) or Talairach reference space; (3) studies conducted on healthy adults. Exclusion criteria: studies conducted on children or the elderly; studies using stimuli with emotional content, reward, or other inhibitory tasks; studies with pharmacological treatment or training; non-standard neuroimaging data analysis or procedures. The selection of these strict criteria allowed us to select homogeneous studies in order to obtain more robust measures [37]. From the pool of potentially eligible studies, we performed further selection in order to identify studies for the comparison (quantitative analysis) between studies on Western vs. Eastern culture matched for the type of experimental paradigm (SST and GoNogo) and participant characteristics (age and education level). See Supplementary Materials (Table S1) for details on culture studies included in the Eastern or Western groups of studies.

### 2.2. Activation Likelihood Estimation (ALE)

Data were analyzed using the Activation Likelihood Estimation (ALE) meta-analysis algorithm implemented in GingerALE 3.0.2 software (www.brainmap.org/ale). ALE is a coordinate-based meta-analysis method that uses peak coordinates from functional studies as inputs, and has been described well in previous methodological papers [38,39,40,41]. The ALE algorithm assesses the convergence of activation foci from different neuroimaging studies, modeled as probability distributions, against null distributions of random spatial associations, while controlling for sample size. To minimize within-experiment effects, the non-additive algorithm described in Turkeltaub et al. [39] was applied. Inference was made at the cluster level, as this provides the best balance between sensitivity and specificity [41]. The cluster-forming threshold was set to *p* < 0.005, and the size of the resulting supra-threshold clusters was compared to a null distribution determined by 2000 permutations of the data (with a threshold of *p* < 0.05). The selected studies are outlined in Supplementary Materials (Table S1).

The first two meta-analyses were performed with the activation foci generated by the Go–Nogo and the SST, and were divided into two (Eastern culture and Western culture) groups of studies. To further examine the shared and differential neural substrates during the inhibition task, the two groups of studies (Eastern culture vs. Western culture) were analyzed. A conjunction analysis was performed to assess the potential overlap among the brain networks from the Western and Eastern culture groups, and a pairwise subtraction analysis was used to identify the differences in activation between these two networks [41]. The analysis was conducted with a statistical threshold of uncorrected *p* < 0.01 and 10,000 permutations, and a cluster-size threshold of 200 mm^3^.

The neuroanatomical coordinates reported in Talairach space [42] were transformed to MNI space for all analyses. Whole-brain maps of the thresholded ALE images were visualized in Mango V.4.0.1 (http://rii.uthscsa.edu/mango/), an anatomical image overlay program, superimposed onto a standardized anatomical template.

## 3. Results

### 3.1. Results of the Study Search

**Figure 1:**
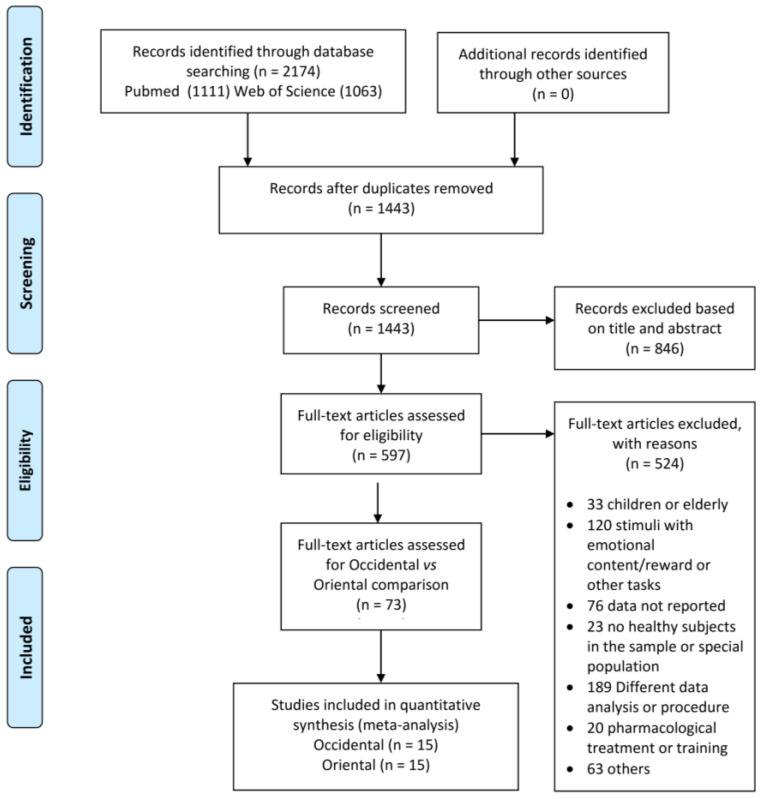
PRISMA flow chart.

A PRISMA flow chart of the article selection process is illustrated in Figure 1. Our search yielded 597 potentially eligible studies. After evaluation of the full text of these articles, 73 studies were potentially eligible. This pool of studies included 14 Eastern and 59 Western studies. In fact, we considered 15 studies from Eastern cultures, because one study (Supplementary Materials, Table S1) presented data with both SST and GoNogo tasks. In order to perform a balanced comparison, a subset of 15 Western studies matching the Eastern group for type of experimental design (SST and GoNogo) and participant characteristics (age and education level) was extracted. Therefore, 30 studies (15 from Eastern culture countries and 15 from Western culture countries) from 2003 to 2022 were included in our quantitative analyses. From these studies, a cumulative number of 719 healthy subjects and 373 foci were obtained. The main characteristics of the studies included in the analysis are reported in the Supplementary Materials (Table S1).

### 3.2. ALE Results

**Figure 2:**
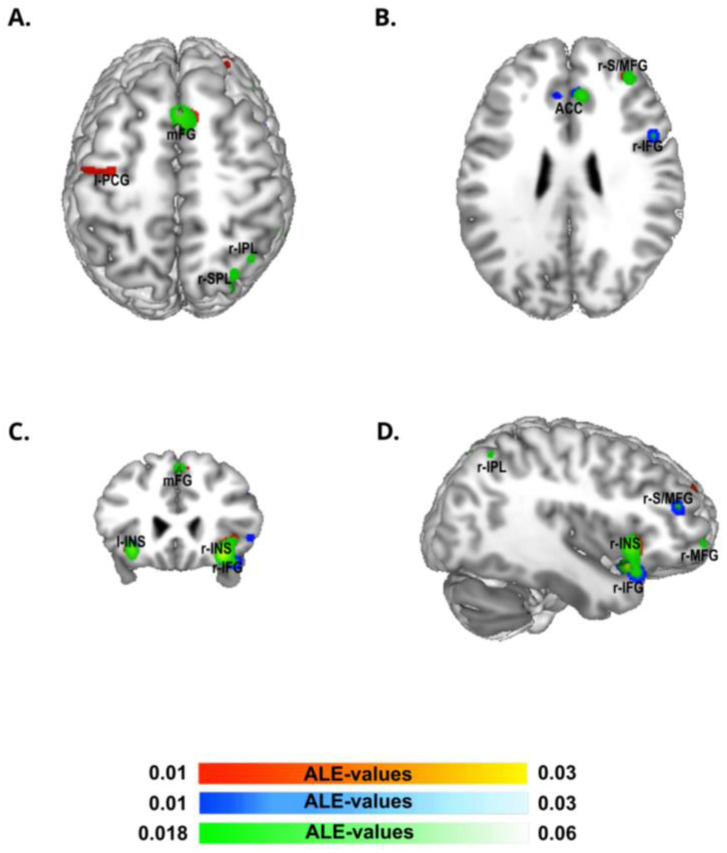
ALE meta-analysis map for the Western and Eastern groups of our data selection. The algorithm converged for the Western group (in yellow-red) on left precentral gyrus (l-PCG), and was bilateral on medial frontal gyrus (mFG), right superior/middle frontal gyrus (r-S/MFG), and right and left insula (r-INS, l-INS). The algorithm converged for Eastern group (in white-blue) on bilateral anterior cingulate cortex (ACC), right inferior frontal gyrus (r-IFG), left striatum nucleus extended to the insula (l-INS), right inferior frontal gyrus (r-IFG) and right superior/middle frontal gyrus (r-S/MFG). The algorithm converged for the conjunction analysis of the two groups (in white-green) bilaterally on medial frontal gyrus (mFG), right inferior parietal lobule (r-IPL), right superior parietal lobule (r-SPL), right superior/middle frontal gyrus (r-S/MFG), bilateral anterior cingulate cortex (ACC), right inferior frontal gyrus (r-IFG), and right (r-INS) and left striatum extended to the insula (l-INS)—*p* < 0.05 cluster-level corrected inference using *p* < 0.005 uncorrected at voxel-level as the cluster-forming threshold.

The ALE meta-analysis of the group of studies from Eastern countries (Figure 2; Table 1) identified the largest cluster size (7408 mm^3^) in the right cerebral hemisphere extending from the striatum, the claustrum, the caudate, the insula, and the inferior frontal gyrus and comprising small portions of the right precentral gyrus, putamen, and globus pallidus. The second cluster (2232 mm^3^) included the right striatum and the claustrum, followed by a cluster (1752 mm^3^) located in the right middle frontal gyrus, a cluster (1640 mm^3^) in the bilateral cingulate gyrus and extending to its anterior part, and, finally, a cluster (1376 mm^3^) in the right middle frontal gyrus.

Regarding the Western group of studies (Figure 2, Table 1), the ALE algorithm converged in a first cluster (4358 mm^3^) in the right cerebral hemisphere, including the striatum, the claustrum, and the right insula and extending to a small part of the right inferior frontal gyrus, followed by an almost symmetric cluster (2152 mm^3^) in the left hemisphere, including the insula and claustrum. The third cluster (2120 mm^3^) was located in the right middle and superior frontal gyrus, whereas the fourth cluster (1768 mm^3^) was centered in the medial prefrontal cortex (pre-SMA). Finally, we observed a last cluster (1496 mm^3^) in the left pre-central gyrus.

As expected, the conjunction analysis between the Western and Eastern groups revealed almost all the above-listed areas (Figure 2, Table S2).

**Figure 3:**
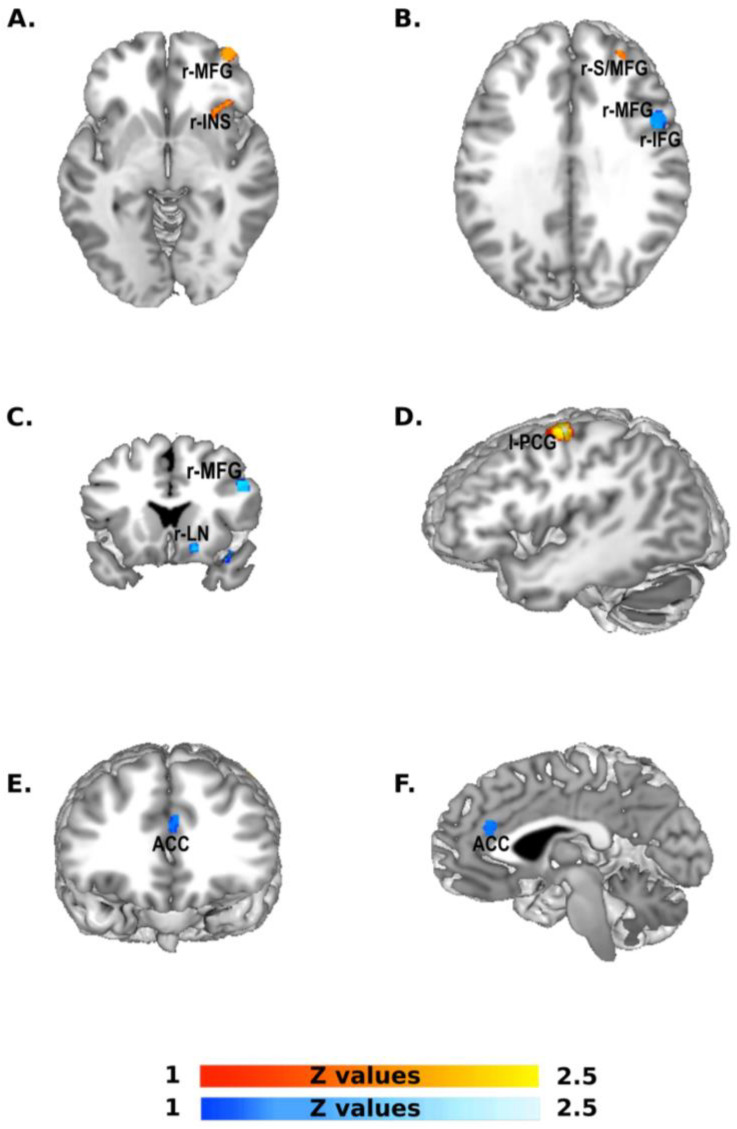
Contrast analysis between Western and Eastern groups. The scale bar in red represents z-values from 1 to 2.5 revealed by the Western > Eastern contrast analysis: left pre-central gyrus (l-PCG), right middle frontal gyrus (r-MFG), right insula (r-INS), right superior frontal gyrus (r-S/MFG). The scale bar in blue represents z-values from 1 to 2.5 revealed by the Eastern > Western contrast analysis: right middle frontal gyrus (r-MFG) and the inferior frontal gyrus (r-IFG), right lentiform nucleus (r-LN).

The contrast analysis (Figure 3, Table 2) between Western and Eastern groups of studies showed, in the former, one cluster (1152 mm^3^) centered in the left pre-central gyrus, followed by a cluster (624 mm^3^) located in the right middle frontal gyrus, with one cluster (352 mm^3^) included in the right insula. Finally, we observed one last cluster (256 mm^3^) in the right superior frontal gyrus.

The opposite contrast (Eastern versus Western) showed a first cluster (632 mm^3^) in the right middle frontal gyrus, the inferior frontal gyrus and the pre-central gyrus, followed by a cluster (312 mm^3^) in the right inferior frontal gyrus and a cluster (296 mm^3^) in the anterior cingulate gyrus. Finally, we observed one last cluster (280 mm^3^) in the striatum.

## 4. Discussion

The present study aimed to explore how belonging to either Western or Eastern cultures may result in the distinct functional recruitment of brain areas involved in cognitive control. To accomplish this, we gathered data from studies investigating cognitive control in Western and Eastern population samples, utilizing fMRI to measure performance in the Stop Signal Task (SST) and Go/Nogo tasks.

Our meta-analyses revealed that most of the clusters identified via ALE in both Western and Eastern culture groups were located in the same brain regions. This was evident not only in the separate analyses of each group of studies, but also in the conjunction analysis, which allowed us to identify common areas of activation across both populations. The regions observed in the present meta-analysis align with those identified in previous meta-analyses of fMRI studies on the Go–Nogo and SST tasks [29,43,44]. Specifically, the pre-supplementary motor area (pre-SMA), right prefrontal cortices (r-IFG, r-MFG, r-SFG), anterior cingulate cortex (ACC), insula, claustrum, and thalamus were consistently observed across these studies.

Notably, upon conducting a contrast analysis on the two groups of studies (Eastern vs. Western culture countries), we observed differences between the underlying networks revealed by the ALE. A discernible pattern of significant clusters was distributed over the right prefrontal cortex. In particular, the contrast between Eastern and Western study groups revealed activation in the right inferior frontal gyrus, while the opposite contrast (Western vs. Eastern study groups) showed distinct clusters in the right middle and superior frontal gyri. These results indicate pronounced recruitment of the middle and superior regions of the right prefrontal cortex in the Western group, whereas the lower regions (i.e., inferior prefrontal cortex) are more prominently activated in the Eastern group. This finding is particularly noteworthy as the prefrontal cortex is widely recognized as a region responsible for the inhibitory control of cognitive processes [45,46,47,48], and there appears to be a dissociation between the right inferior frontal gyrus and the middle/superior frontal gyri depending on the inhibitory phase. To be more precise, the right inferior frontal gyrus (r-IFG) plays a significant role in the attentional aspects of cognitive control and is involved in all stages of inhibition. However, its primary function, according to recent studies, lies in the proactive phase of inhibition [29,49,50]. That is, a top-down form of inhibitory control that governs the processes preceding event occurrence, thereby improving the accuracy and efficiency of motor responses [20,51]. In contrast, the right superior and middle frontal gyri (r-S/MFG) are pivotal brain regions for reactive inhibition, which is a bottom-up form of inhibitory control. Reactive inhibition is believed to act as a “cut-trigger,” halting an already initiated motor response [29,49,50,52].

The results herein highlight the greater propensity of Eastern populations to activate brain regions that mediate proactive inhibitory control, while those from Western cultures may rely more on reactive inhibitory brain regions to achieve accurate inhibition during cognitive control tasks. This is an intriguing result as it may suggest that proactive inhibition, as reflected in brain activations, may play a crucial role in fostering the collective and interdependent behavior that is commonly observed in people of Eastern cultures. On the other hand, reactive inhibition, as a cognitive control mechanism, may be predominant in achieving efficient cognitive control for cultures that prioritize individualism and independence, which are commonly observed in Western cultures [3,4,5,6,7].

However, from an alternative perspective, we can speculate that the difference in prioritizing reactive control might arise from the Western inclination for swift action, influenced by their fast-paced lifestyles, while the Eastern mindset might tend to favor contemplation and a more meditative approach to life.

Another difference observed in the contrast between the Eastern and Western groups of studies is the anterior cingulate cortex. This result corroborates our interpretation; in fact, the anterior cingulate cortex is not just a brain region responsible for detecting and resolving conflict [53,54,55], but it is also at the basis of the proactive mechanism, as recently pointed out in different studies [48,50]. Indeed, being able to better manage and resolve conflicts is undoubtedly a necessary skill for adapting to more collective cultures such as those in the Eastern countries. Hence, it is not surprising that this brain region has been consistently identified as neuro-anatomically and functionally [56] different in individuals from Eastern cultures when compared to their Western counterparts.

Finally, we observed greater activation of the striatum in the group of Eastern culture countries and higher activation of the left precentral cortex in the group of Western culture countries. The former finding aligns with the possibility that individuals from Eastern cultures may possess stronger inhibitory control, although this assertion lacks conclusive evidence. Conversely, the latter finding suggests that individuals from Western cultures may exhibit a more impulsive response during the Go–Nogo and SST tasks. Indeed, Gavazzi et al. [48] reported a positive correlation between impulsivity during the proactive phase of a Go–Nogo task and increased BOLD signals in the left precentral gyrus. This is a result that is consistent with many studies reporting that the left hemisphere is crucial in the link between impulsivity and inhibitory control at the functional and morphometric neuroimaging levels [57]. Additionally, there is evidence showing that the left hemisphere may be dominant, or is at least involved, in some aspects of inhibitory control [58,59]. Despite the existence of several pieces of evidence linking inhibition and impulsivity, an ongoing debate remains regarding the possibility [60] that impulsivity may not be directly related to inhibitory control. Therefore, future studies are needed to assess our interpretation of the precentral gyrus.

Taking into account the examination of these findings in the broader ongoing discussion about the complex interplay between culture and the brain, and their reciprocal influence [61], it is plausible to speculate that environmental factors significantly contribute to the impact of culture on inhibitory neuroprocesses. Notably, the cultural differentiation of the brain appears to be influenced more by the cultural aspects of the environment in which individuals reside, rather than solely by their race or original culture [62]. However, it is important to note that the involvement of genetic or gene–environment effects cannot be completely discounted. In fact, genetic expression appears to be implicated in cultural aspects, such as the acquisition of cultural norms [63].

Overall, the present findings suggest differential activation of the inhibitory network in Eastern-collectivist and Western-individualist cultures. Our meta-analysis corroborates the findings of the few studies conducted to compare inhibitory processes using a cross-cultural design [62,64,65]. Namely, differential activations of brain regions embedded in the inhibitory network (i.e., the dACC in relation to prediction error [64] and the IFG [62,65]) have been reported. Therefore, previous neuroimaging data may support our hypothesis of higher engagement of the proactivity-related brain regions [29] during inhibitory tasks in collectivist cultures compared to individualist cultures. Interestingly, Telzer et al. [65] found a greater decline in inhibitory behavioral performance over time in Western participants than in Eastern participants, an effect paralleled by increasing activation of the IFG among the collectivist group, while this neural activation stayed low in the individualist group. Most evidence of cultural differentiation in inhibitory behavioral performance comes from childhood studies, with children from collectivist cultures outperforming those from individualist ones in inhibition efficiency [6,14,66,67,68,69,70]. Regarding adults, if neural results always point out a cultural differentiation, this is not always the case for behavioral data. Namely, enhanced inhibitory performance for collectivism vs. individualism have been found in some studies [65,71], but not in others [62,64,72]. Different task designs or group compositions might represent confounding variables to be taken into careful consideration while interpreting contrasting evidence from these behavioral findings. A dissociation between neuroimaging and behavioral data does not come as a surprise, as behavioral measures, especially when embedded in neuroimaging designs, might lack sensibility. In fact, cultural differentiation in neurocognitive functions might occur even in the absence of purely behavioral evidence thanks to the extra fine-grained level of analysis [62,73,74].

Some limitations should be acknowledged. First, to reach adequate statistical power, we were forced to use broad inclusion criteria for studies examining subjects from Western and Eastern culture countries. In addition, our current methodology did not enable us to eliminate certain potential confounding variables that require further examination in future studies. Specifically, two key factors that necessitate exploration in subsequent research are the lack of measures allowing for the exploration of brain–behavior relationships, and the requirement for a quantitative assessment of the cultural aspects pertaining to the participants included in the studies. Additionally, due to missing data, we were unable to retrieve education information from all of the studies. Finally, it is important to recognize that our meta-analysis did not reveal some commonly observed subcortical brain areas, such as the subthalamic nucleus [75,76]. This limitation has been commonly observed in fMRI studies, as demonstrated by Isherwood et al. [44].

Future studies should aim to utilize larger and more diverse samples, control for more confounding variables, and incorporate culturally sensitive experimental designs to elucidate the mechanisms underlying these cultural differences in inhibitory control and the impact of cultural context on the development and manifestation of these processes. Moreover, understanding how these cultural differences in inhibitory control influence behavior and cognition can have practical implications, such as in educational and clinical settings. For instance, in the case of clinical settings, this knowledge may help to develop customized interventions that align with the cultural context of patients, thereby increasing the likelihood of positive outcomes, enhancing therapeutic alliances, and improving treatment adherence [77,78]. Furthermore, it can aid in identifying at-risk individuals and implementing early interventions to prevent mental health problems.

By better understanding the neural mechanisms associated with cultural differences, we can gain insights into how culture influences cognition and behavior, and ultimately develop more effective interventions to address cultural disparities in health and well-being.

## Figures and Tables

**Table 1:** Results of ALE meta-analysis. Foci are reported as MNI coordinates. BA = Broadman Area.

Eastern Group of Studies: ALE Meta-Analysis Computed from Our Study Selection						
Cluster	x	y	z	ALE	*p*	Label (Nearest Gray Matter within 5 mm)
1	32	16	−12	0.02397523	8.957319 × 10^−9^	Right Cerebrum. Sub-lobar. Claustrum. Gray Matter
	16	16	−8	0.016835388	3.894887 × 10^−6^	Right Cerebrum. Sub-lobar. Caudate. Gray Matter. Caudate Head
	38	16	−2	0.016307237	5.931126 × 10^−6^	Right Cerebrum. Sub-lobar. Claustrum. Gray Matter
	50	18	2	0.014818636	2.2523935 × 10^−5^	Right Cerebrum. Frontal Lobe. Precentral Gyrus. Gray Matter. BA 44
	56	16	14	0.01302748	1.0037824 × 10^−4^	Right Cerebrum. Frontal Lobe. Inferior Frontal Gyrus. Gray Matter BA 44
	22	6	−6	0.0101995105	6.4743846 × 10^−4^	Right Cerebrum. Sub-lobar. Lentiform Nucleus. Gray Matter. Putamen
	16	2	−2	0.009658105	8.880825 × 10^−4^	Right Cerebrum. Sub-lobar. Lentiform Nucleus. Gray Matter. Medial Globus Pallidus
2	−30	18	−4	0.018294685	1.2900151 × 10^−6^	Left Cerebrum. Sub-lobar. Claustrum. Gray Matter
	−26	10	0	0.01170183	2.4654568 × 10^−4^	Left Cerebrum. Sub-lobar. Lentiform Nucleus. Gray Matter. Putamen
3	48	10	28	0.016780104	4.072827 × 10^−6^	Right Cerebrum. Frontal Lobe. Inferior Frontal Gyrus. Gray Matter. BA 9
	48	16	32	0.01458163	2.7073333 × 10^−5^	Right Cerebrum. Frontal Lobe. Middle Frontal Gyrus. Gray Matter. BA 9
	44	4	44	0.009647945	8.9332176 × 10^−4^	Right Cerebrum. Frontal Lobe. Middle Frontal Gyrus. Gray Matter. BA 6
	44	10	42	0.009213654	0.001171954	Right Cerebrum. Frontal Lobe. Middle Frontal Gyrus. Gray Matter. BA 6
4	−6	32	22	0.017859418	1.7803774 × 10^−6^	Left Cerebrum. Limbic Lobe. Anterior Cingulate. Gray Matter. BA 32
	6	34	24	0.01445027	3.053667 × 10^−5^	Right Cerebrum. Limbic Lobe. Cingulate Gyrus. Gray Matter. BA 32
	−8	30	32	0.009196116	0.0011785898	Left Cerebrum. Frontal Lobe. Cingulate Gyrus. Gray Matter. BA 32
5	36	40	20	0.021027796	1.342605 × 10^−7^	Right Cerebrum. Frontal Lobe. Middle Frontal Gyrus. Gray Matter. BA 9
Western Group of Studies: ALE Meta-Analysis Computed from Our Study Selection						
Cluster	x	y	z	ALE	*p*	Label (Nearest Gray Matter within 5 mm)
1	32	20	−8	0.036751416	1.1696465 × 10^−12^	Right Cerebrum. Sub-lobar. Claustrum. Gray Matter
2	−30	20	−8	0.021623248	3.6322265 × 10^−7^	Left Cerebrum. Sub-lobar. Claustrum. Gray Matter
	−44	20	0	0.010548196	8.794995 × 10^−4^	Left Cerebrum. Sub-lobar. Insula. Gray Matter. BA 13
	−28	30	0	0.009875406	0.0013448054	Left Cerebrum. Sub-lobar. Insula. Gray Matter. BA 13
3	32	46	16	0.01598811	2.3766925 × 10^−5^	Right Cerebrum. Frontal Lobe. Middle Frontal Gyrus. Gray Matter. BA 10
	32	42	24	0.01520087	4.282069 × 10^−5^	Right Cerebrum. Frontal Lobe. Middle Frontal Gyrus. Gray Matter. BA 9
	28	50	30	0.014807648	5.7067977 × 10^−5^	Right Cerebrum. Frontal Lobe. Superior Frontal Gyrus. Gray Matter. BA 9
4	4	16	50	0.018120997	4.6963864 × 10^−6^	Right Cerebrum. Frontal Lobe. Superior Frontal Gyrus. Gray Matter. BA 6
5	−44	−10	52	0.013735037	1.20725206 × 10^−4^	Left Cerebrum. Frontal Lobe. Precentral Gyrus. Gray Matter. BA 4
	−34	−10	50	0.013622271	1.3100117 × 10^−4^	Left Cerebrum. Frontal Lobe. Precentral Gyrus. Gray Matter. BA 4
	−44	−2	54	0.010301465	0.0010217617	Left Cerebrum. Frontal Lobe. Precentral Gyrus. Gray Matter. BA 6

**Table 2:** Results of ALE contrast analysis between Western and Eastern studies. MNI coordinates. BA = Broadman Area.

Eastern–Western Contrast: ALE Meta-Analysis Computed from Our Study Selection						
Cluster	x	y	z	ALE	*p*	Label (Nearest Gray Matter within 5 mm)
1	52	16	32	0.0179	2.0991917	Right Cerebrum. Frontal Lobe. Middle Frontal Gyrus. Gray Matter. BA 9
	44	14	32	0.0186	2.0835624	Right Cerebrum. Frontal Lobe. Precentral Gyrus. Gray Matter. BA 9
	50	6	30	0.0195	2.0641868	Right Cerebrum. Frontal Lobe. Inferior Frontal Gyrus. Gray Matter. BA 6
	46	6	30	0.0197	2.0599847	Right Cerebrum. Frontal Lobe. Precentral Gyrus. Gray Matter. BA 6
	50.7	12.7	28	0.0294	1.8896858	Right Cerebrum. Frontal Lobe. Inferior Frontal Gyrus. Gray Matter. BA 9
	44	14	28	0.0304	1.8749471	Right Cerebrum. Frontal Lobe. Inferior Frontal Gyrus. Gray Matter. BA 9
2	36	14	−18	0.0149	2.1727386	Right Cerebrum. Frontal Lobe. Inferior Frontal Gyrus. Gray Matter. BA 13
3	−2.4	33.4	24.8	0.0386	1.7671685	Left Cerebrum. Limbic Lobe. Cingulate Gyrus. Gray Matter. BA 32
	−2	31.7	19.3	0.0444	1.701762	Left Cerebrum. Limbic Lobe. Anterior Cingulate. Gray Matter. BA 24
4	14	14	−12	0.0206	2.0415115	Right Cerebrum. Sub-lobar. Caudate. Gray Matter. Caudate Head
	20	8	−8	0.0369	1.7878513	Right Cerebrum. Sub-lobar. Lentiform Nucleus. Gray Matter. Putamen
Western–Eastern Contrast: ALE Meta-Analysis Computed from Our Study Selection						
Cluster	x	y	z	ALE	*p*	Label (Nearest Gray Matter within 5 mm)
1	−41.7	−9.5	53.5	0.0056	2.536396	Left Cerebrum. Frontal Lobe. Precentral Gyrus. Gray Matter. BA 4
	−45	0	54	0.0059	2.5180695	Left Cerebrum. Frontal Lobe. Precentral Gyrus. Gray Matter. BA 6
	−40	−14	50	0.0116	2.270125	Left Cerebrum. Frontal Lobe. Precentral Gyrus. Gray Matter. BA 4
	−46.9	−7.7	53.9	0.0118	2.2635794	Left Cerebrum. Frontal Lobe. Precentral Gyrus. Gray Matter. BA 4
	−36	−12	48	0.0245	1.9685917	Left Cerebrum. Frontal Lobe. Precentral Gyrus. Gray Matter. BA 4
	−46	−14	50	0.0253	1.9548566	Left Cerebrum. Parietal Lobe. Postcentral Gyrus. Gray Matter. BA 3
	−32	−12	52	0.0376	1.7792426	Left Cerebrum. Frontal Lobe. Precentral Gyrus. Gray Matter. BA 4
2	38	50	−2	0.0138	2.2029254	Right Cerebrum. Frontal Lobe. Middle Frontal Gyrus. Gray Matter. BA 10
	37	58	1	0.0231	1.9935615	Right Cerebrum. Frontal Lobe. Middle Frontal Gyrus. Gray Matter. BA 10
	35.3	54	−1	0.0254	1.9531654	Right Cerebrum. Frontal Lobe. Middle Frontal Gyrus. Gray Matter. BA 10
3	38	24	−2	0.0103	2.3152363	Right Cerebrum. Sub-lobar. Insula. Gray Matter. BA 13
	32	20	−2	0.0196	2.0620813	Right Cerebrum. Sub-lobar. Claustrum. Gray Matter
	26	16	−2	0.0251	1.9582559	Right Cerebrum. Sub-lobar. Lentiform Nucleus. Gray Matter. Putamen
4	24	48	30	0.0261	1.9414806	Right Cerebrum. Frontal Lobe. Superior Frontal Gyrus. Gray Matter. BA 9

## Data Availability

Data are available from the corresponding author upon request.

## Supplementary Materials

The following supporting information can be downloaded at: https://www.mdpi.com/article/10.3390/brainsci13060907/s1, Table S1: Characteristics of studies included in the quantitative analysis; Table S2: Eastern–Western and Western-Eastern contrast analyses: ALE meta-analysis computed from our study selection.
